# WNT signaling in neuronal maturation and synaptogenesis

**DOI:** 10.3389/fncel.2013.00103

**Published:** 2013-07-04

**Authors:** Silvana B. Rosso, Nibaldo C. Inestrosa

**Affiliations:** ^1^Laboratorio de Toxicología Experimental, Facultad de Ciencias Bioquímicas y Farmacéuticas, Universidad Nacional de RosarioRosario, Santa Fe, Argentina; ^2^Centro de Envejecimiento y Regeneración, Departamento de Biología Celular y Molecular, Facultad de Ciencias Biológicas, Pontificia Universidad Católica de ChileSantiago, Chile

**Keywords:** Wnt factors, neuronal development, dendrite, synapses, Alzheimer disease

## Abstract

The Wnt signaling pathway plays a role in the development of the central nervous system and growing evidence indicates that Wnts also regulates the structure and function of the adult nervous system. Wnt components are key regulators of a variety of developmental processes, including embryonic patterning, cell specification, and cell polarity. In the nervous system, Wnt signaling also regulates the formation and function of neuronal circuits by controlling neuronal differentiation, axon outgrowth and guidance, dendrite development, synaptic function, and neuronal plasticity. Wnt factors can signal through three very well characterized cascades: canonical or β-catenin pathway, planar cell polarity pathway and calcium pathway that control different processes. However, divergent downstream cascades have been identified to control neuronal morphogenesis. In the nervous system, the expression of Wnt proteins is a highly controlled process. In addition, deregulation of Wnt signaling has been associated with neurodegenerative diseases. Here, we will review different aspects of neuronal and dendrite maturation, including spinogenesis and synaptogenesis. Finally, the role of Wnt pathway components on Alzheimer’s disease will be revised.

## INTRODUCTION

The development and specification of different organs and tissues that take place during embryonic development is tightly coordinated by specific signaling molecules known as morphogens including sonic hedgehog (Shh), transforming growth factor β (TGF β), fibroblast growth factor (FGF), and Wnt factors. These ligands bind to specific receptors and activate intracellular cascades that regulate different cellular processes and behavior (Tabata and Takei, 2004).

**FIGURE 1:**
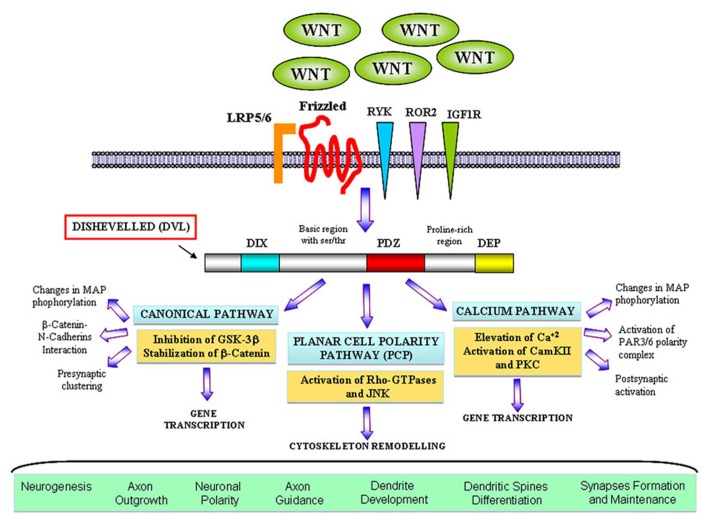
**The main branches ofWnt signalling pathways in neuronal development.** Wnt factors bind to their classical receptor Frizzleds (Fz) or interact with tyrosine kinase receptors such as RYK, ROR2, and also IGF1R depending on the cellular context. In the *canonical or WNT/β-catenin pathway* Wnt also interacts with the LRP5/6 co-receptor and activates Dvl that contains three domains involving in different neuronal functions. Activation of Dvl mainly results in the GSK3β inhibition and the β-catenin accumulation in the cytosol which translocates to the nucleus where it activates specific gene transcription. Divergent pathways have been shown to control axon and dendrite morphology and presynaptic function. In the *planar cell polarity (PCP) pathway* Wnt binds to Fz and activates Dvl, which in turns signals to small Rho-GTPases proteins. Activation of Rac induces changes in the activity of JNK leading to changes in the cytoskeleton. This cascade was implied in dendrite development and complexity. In the *Wnt/calcium pathway*, activation of Dvl induces the increase of intracellular level of calcium and activation of PKC and CamKII affecting the transcription of NF-AT nuclear factor. However, divergent cascades involving the activation of CamKII have been identified to modulate neuronal polarity, dendritic spines morphology and synapses.

Wnt factors are secreted morphogens that regulate cell fate decision, cell polarity, and embryonic patterning (Parr and McMahon, 1994; Wodarz and Nusse, 1998; Nusse and Varmus, 2012). In the last years, a great body of evidence has demonstrated that Wnt signaling also plays a key role in the formation and modulation of neuronal circuits. In the nervous system, Wnt proteins are required during development for early patterning by acting as posteriorising signals, for neural crest cell induction, neural precursor, cell proliferation, and neurogenesis (Ciani and Salinas, 2005; Toledo et al., 2008). Moreover, Wnts regulate essential processes including neuronal migration, neuronal polarization, axon guidance, dendrite development, and synapse formation which are required for a proper brain wiring (Ciani and Salinas, 2005; Inestrosa and Arenas, 2010). Wnts are highly conserved molecules among animal species (van Amerongen and Nusse, 2009). Wnt proteins bind to the amino-terminal cysteine-rich domain (CRD) of the seven transmembrane Frizzled (Fz) receptors. In addition to Fz, Wnt ligands can also signal through other receptors with tyrosine kinase activity such as atypical receptor related tyrosine kinase (RYK) and orphan receptor tyrosine kinase (ROR2) (Inoue et al., 2004; Logan and Nusse, 2004). Interestingly, insulin-like growth factor 1 (IGF-1) receptor (IGF-1r) is emerging as a potential Wnt receptor to modulate neuronal events (Hu et al., 2012). After Wnts bind to their receptors, they activate a number of possible intracellular cascades: the Wnt–β-catenin pathway (canonical pathway), the planar cell polarity pathway (PCP pathway) and the calcium pathway (**Figure 1**). In the canonical pathway, the binding of the Wnt ligand to its Fz receptor and the low-density lipoprotein receptor-related protein 5/6 (LRP5/6) activate the scaffolding protein Dishevelled (Dvl), which in turn induces the disassembly of the complex formed by axin, adenomatous polyposis coli (APC), and the serine/threonine kinase glycogen synthase kinase-3β (GSK-3β). In the absence of Wnt signaling, β-catenin is synthesized but rapidly degraded due the phosphorylation by GSK-3β (Orford et al., 1997; Salic et al., 2000). In the presence of Wnt, GSK-3β is inhibited leading to the accumulation and the stabilization of β-catenin in the cytosol and its translocation into the nucleus, where it associates with the transcription factor TCF (T cell factor)/LEF (lymphoid enhancing factor) to regulate Wnt target genes (Gordon and Nusse, 2006). In the PCP pathway, Wnt binds to its receptor and activates Dvl which leads to changes in both actin and microtubule reorganization. These alterations involve the activation of the small Rho-GTPases proteins and c-Jun-N-terminal kinase (JNK; Rosso et al., 2005; Gordon and Nusse, 2006). Finally, in the calcium pathway, Wnt increases the intracellular level of Ca^2+^ leading to the activation of calcium sensitive kinases such as protein kinase C (PKC) and Ca^2+^/calmodulin-dependent protein kinase II (CamKII) and the nuclear translocation of the transcription factor nuclear factor of activated T cells (NFACT).

Importantly, the same ligand can function through different Wnt pathway depending on the receptor and cellular context. In this review, we will discuss the role of Wnt proteins during neuronal development and maturation. Firstly, we will describe the Wnts function during initial neuronal differentiation and axon behavior. Then, we will focus on the role of Wnt factors on neuronal maturation particularly the formation of dendritic arbors and their function as modulators of synaptic physiology. Finally, we will concentrate on the preventive role of Wnt signaling on neurodegenerative diseases in particular Alzheimer’s disease (AD).

## Wnt SIGNALING AND NEURONAL DEVELOPMENT

### Wnt REGULATE AXON OUTGROWTH AND MORPHOLOGY

The proper function of the nervous system depends on the morphological complexity of the neurons, the participation of non-neuronal cells and the establishment of suitable neuronal connections. Neurons are highly polarized cells that ensure an unidirectional flow of information. After they are born, neurons differentiate and establish two compartments: axon and dendrites which have distinct molecular composition, morphology and functioning (see Dotti et al., 1988). This polarized arrangement is fundamental for neuronal function in order to receive and propagate electrical signals to distinct sites.

A very useful system for studying neuronal polarization and axon growth is the cultured hippocampal neurons, which displays five developmental stages. Firstly, neurons elaborate lamellipodia (stage 1) and then, short neurites or minor processes (stage 2). After that, one of these neurites grows faster and becomes the axon with a large and highly dynamic growth cone (stage 3), leaving the rest of the neurites to form dendrites (stage 4). Finally, dendrite maturation takes place and very complex dendritic arbors are able to initiate synaptic function (stage 5; Dotti et al., 1988; Craig and Banker, 1994).

Neuronal polarization and maturation are controlled not only by intrinsic factors and genes expression programs but also by molecules which come from the extracellular matrix. These extrinsic molecules are potential regulators for axon specification and pathfinding, neuronal maturation and synapses formation and maintenance. Among these molecules there are neurotrophic factors such as nerve growth factor (NGF), brain-derived neurotrophic factor (BDNF), and neurotrophin 3 (NT3) which function as neuronal polarity and axon development modulators through the activation of tyrosine receptor kinases (Trks; Huang and Reichardt, 2003). However, another family of extracellular factors called Wnt proteins is emerging as a regulator of initial axon differentiation, growth and axonal behavior. Interestingly, Wnt5a is required for NGF-dependent axonal branching and growth (Bodmer et al., 2009). Wnt5a would function as a key NGF downstream effector in developing sympathetic neurons by locally activation of PKC (Bodmer et al., 2009). In addition, NGF enhances the expression of Wnt5a in sympathetic neurons. Furthermore, Wnt5a-null mice neurons show deficits in NGF-dependent axonal development (Bodmer et al., 2009). Another study carried out in *Drosophila* demonstrates that Wnt5a functions as a ligand in the PCP pathway during axonal growth and branching (Shimizu et al., 2011). Importantly, other PCP pathway components such as Fz, strabismus, flamingo, and disheveled are cooperatively required for axonal targeting and branching. Authors propose that Wnt5a and the PCP pathway regulate axonal development in *Drosophila* neurons carefully (Shimizu et al., 2011).

Disheveled is the first downstream effector of Wnt signaling pathways. Many studies have demonstrated that Dvl is a neurite growth and differentiation key regulator. Dvl expression in neuroblastoma 2A cell (N2A cells) promotes neurite outgrowth and induces N2A cells differentiation (Fan et al., 2004). This neuronal remodeling depends on a Dvl N-terminal DVL domain (DIX). The DIX domain is essential for the Dvl effect on neuronally differentiating N2A cells (Fan et al., 2004). Accordingly, other studies have shown that Dvl regulates neurite extension through the microtubule stability regulation. Dvl colocalizes with axonal microtubules and protects stable microtubules from nocodazole depolymerization (Krylova et al., 2000; Rosso et al., 2005). Dvl increases microtubule stability through GSK-3β inhibition and changes in the microtubule-associated protein 1B (MAP1B) activity (Ciani et al., 2004). Additionally, another work reveals that Dvl promotes axon differentiation by regulating atypical PKC (aPKC; Zhang et al., 2007). In cultured hippocampal neurons aPKC is directly regulated by Dvl. Thus, Dvl downregulation abolishes axon differentiation. In contrast, Dvl overexpression induces multiple axons formation (Zhang et al., 2007). Interestingly, the authors show that Dvl associates and activates aPKC in these neurons and the expression of a aPKC dominant negative prevents the Dvl multiple axons formation (Zhang et al., 2007). To add, Dvl forms a complex with PAR3, PAR6, and aPKC, resulting in aPKC stabilization and activation. Furthermore, treatment with Wnt5a, a non-canonical Wnt factor, induces the aPKC activation and promotes axon differentiation in cultured hippocampal neurons (Zhang et al., 2007). These evidences demonstrate the Wnt pathways participation on the initial neuronal differentiation.

Several Wnt signaling effectors are very well known as axonal modulators, such as GSK3β and APC belonging to the canonical pathway and the small Rho-GTPases proteins and associated kinases from the PCP pathway. Many proteins regulate the cytoskeleton dynamics and organization functioning as direct targets of GSK-3β. For example, microtubule-associated proteins (MAPs), such as MAP1B, tau, and MAP2, which are expressed in developing neurons, function as microtubule stabilizers and can be phosphorylated by GSK-3β (Berling et al., 1994; Lucas et al., 1998). Another protein that is directly phosphorylated by GSK-3β is APC, an important player in the Wnt signaling pathway and a microtubule plus-end binding protein that is accumulated at growth cones (Zumbrunn et al., 2001; Zhou et al., 2004). Phosphorylation of these proteins by GSK-3β changes their ability to bind microtubules and their function as modulators of microtubule dynamics (Gonzalez-Billault et al., 2004; Zhou et al., 2004; Baas and Qiang, 2005). Thus, changes in the cytoskeletal proteins phosphorylation might contribute to axon determination and growth. In addition, it has been observed that cultured neurons exposed to Wnts elicits an axonal remodeling, a process characterized by axonal spreading and growth cone enlargement (Hall et al., 2000; Krylova et al., 2002). In the presence of Wnt, microtubules form loops as observed in axonal growth cones of granule cells (Hall et al., 2000). This microtubule reorganization is likely to determine changes in axon behavior. Importantly, loss of Wnt7a or its effector DVL causes severe defects in the terminal remodeling of axons *in vivo* (Ahmad-Annuar et al., 2006; Hall et al., 2000). According to this evidence, another later study showed that exposure to Wnt3a decreases the speed of growth cone advance whilst increasing growth cone size (Purro et al., 2008). The authors propose that Wnt regulates axon behavior through changes in microtubule growth directionality induced by a decrease in the APC level on microtubule plus-ends (Purro et al., 2008).

Taken together, these data clearly demonstrate that Wnt factors and their effectors positively modulate axon outgrowth and growth cone behavior through changes in the cytoskeletal components activity affecting their organization and stability.

### Wnt PROTEINS CONTROL DENDRITIC MORPHOGENESIS

A mature neuron elicits a clear architecture characterized by a long distally branched axon which transmits signals, and a very complex dendritic arbor which is specialized to collect and integrate signals. Appropriate connections within the nervous system require the establishment of a polarized morphology to ensure unidirectional signal propagation (Jan and Jan, 2003; Conde and Caceres, 2009). Although a proper dendrite remodeling and shape underlies the normal mammalian brain function, including cognition and memory formation, abnormal dendritic development closely correlates with mental retardation and a number of central nervous system (CNS) disorders including Down’s, Rett, and Fragile X syndromes (Comery et al., 1997; Kaufmann and Moser, 2000; Miller and Kaplan, 2003; De Rubeis and Bagni, 2010).

The molecular and cellular mechanisms that regulate dendritic growth and refinement are an area of intense research. Each neuron acquires its precise dendritic pattern through the regulation of its cytoskeleton by activating signaling pathways that change the activity, localization, and stability of cytoskeletal regulators (Conde and Caceres, 2009). Dendrite growth is a very dynamic process and the pattern of dendritic trees is believed to be regulated by an interplay between an intrinsic genetic program, extrinsic factors, and neuronal activity (Cline, 2001; Scott and Luo, 2001; Whitford et al., 2002a; Jan and Jan, 2003). Many extracellular factors have been identified as regulators of dendritic growth and branching. For example, in vertebrates NT3, BDNF, and NGF can function as extrinsic factors to modulate the neuronal dendritic morphology (McAllister et al., 1995). Furthermore, the same neurotrophic factor can either inhibit or promote dendritic outgrowth depending on the type of neuron (McAllister et al., 1997). Semaphorin-3A (Sema3A) acts through the neuropilin-1 receptor, functions as chemoattractant factor for growing apical dendrites whereas antibody against neuropilin-1 can attenuate this dendrite orientation (Polleux et al., 2000). Consistent with this notion, in Sema3A null mice, many of the cortical pyramidal neurons have misoriented apical dendrites. Several evidences have shown that other extracellular factors such as Notch1 and Slit1 also regulate the emergence and growth of basal dendrites (Threadgill et al., 1997; Redmond et al., 2000; Whitford et al., 2002b). In addition, bone morphogenetic protein 7 (BMP7), a member of the TGF-β superfamily of ligands that are crucial for nervous system development, also affect dendritic morphogenesis in cultured neurons (Whitford et al., 2002a; Miller and Kaplan, 2003; Podkowa et al., 2010).

**FIGURE 2:**
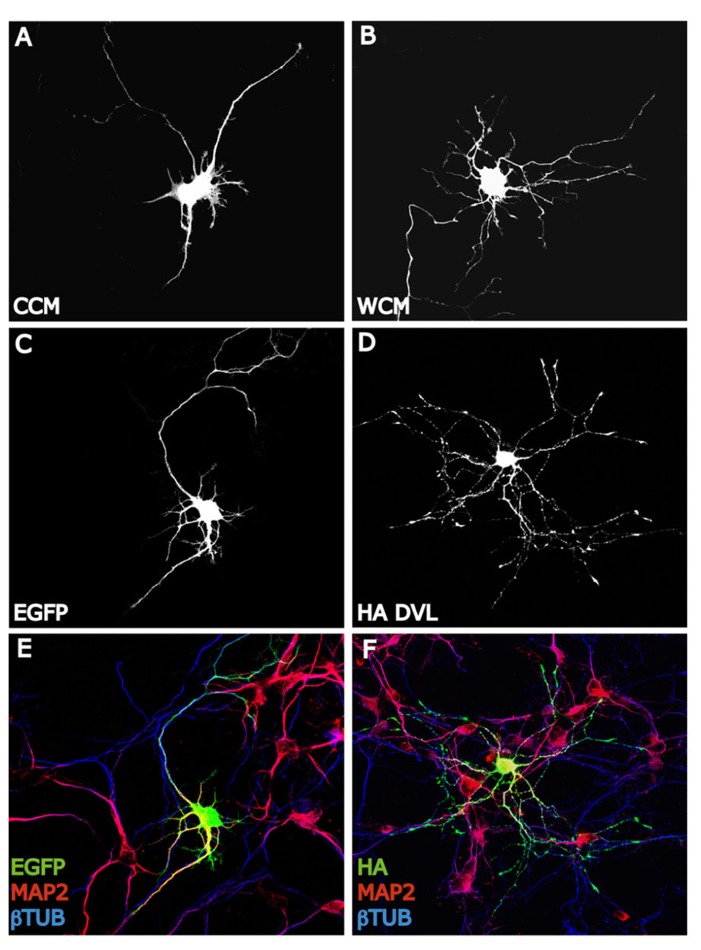
**Wnt-Dvl pathway modulates dendrite development.** **(A,B)** EGFP expressing hippocampal neurons of 3 days *in vitro* (DIV) expose to Wnt7b conditioned medium (WCM) show longer and more complex dendritic arborizations compare to EGFP-control cells (CCM). Neurons are staining with GFP antibody to analyze the entire morphology. **(C–F)**: DVL mimics the dendritogenic effect of Wnt7b. EGFP expressing neurons of 3 DIV develop several short and unbranched dendrites (MAP2+) **(C,E)**. However, hippocampal neurons expressing HA-DVL reveal very complex dendritic trees with longer and more number of dendritic branches (MAP2+) **(D,F)**.

Another family of proteins that have been extensively involved in dendrite formation, maintenance, and functioning during the last decade is Wnt proteins. Several evidences suggest that Wnt factors modulate axon morphology and branching probably because they affect the activity and localization of many cytoskeletal regulators. These findings lead to consider that Wnt signaling may also regulate the dendritic trees morphology. Several studies have shown that Wnt proteins regulate dendritic architecture through the activation of different cascades. In this context, Yu and Malenka (2003) have postulated to β-catenin as a critical mediator of dendritic morphology. Thus, overexpression of β-catenin increases dendritic arborization in hippocampal neurons through a non-transcriptional mechanism (Yu and Malenka, 2003). Instead, constitutively active β-catenin increases dendritic arborization through its interaction with N-cadherin and αN-catenin (neural-catenin). Conversely, sequestering endogenous β-catenin leads to a decrease in dendritic complexity caused by neural activity (high K+ depolarization) suggesting that the level of endogenous β-catenin is important to the regulation of dendritic branching (Yu and Malenka, 2003). In addition, dickkopf-1 (Dkk-1), an extracellular Wnt antagonist (Glinka et al., 1998), blocks the dendritogenic effect of depolarization by high K^+^ suggesting that neuronal activity regulates Wnt expression or release, which in turn modulates dendritic arborization. Importantly, conditioned medium from depolarized neurons contain higher levels of Wnt than does media from non-stimulated cells (Yu and Malenka, 2003). Another study showed that neuronal activity enhances the expression of Wnt-2, which stimulates dendritic complexity in cultured hippocampal neurons (Wayman et al., 2006). Activity-dependent dendritic outgrowth and branching in cultured neurons and slices is mediated through activation of Ca^+2^ depending signaling pathway (Wayman et al., 2006). In agreement with these studies, it has been demonstrated that Wnt signaling through Dvl stimulates dendritic growth and branching in hippocampal neurons (Rosso et al., 2005). Particularly, Wnt7b which is expressed in the hippocampus during dendritogenesis increases dendritic arborization by increasing dendritic length and the formation of complex branches. This effect is blocked by a Wnt scavenger, Sfrp1 (soluble Fz-related protein-1). Sfrp1 blocks endogenous Wnt activity present in hippocampal cultures that contributes to the normal dendritic development (Rosso et al., 2005). The Wnt effect on dendrite development is mimicked by Dvl that localizes along the neurites associated with microtubules and is highly concentrated in the peripheral region of growth cones co-localizing with actin cytoskeleton (Rosso et al., 2005; **Figure 2**). Dvl1 mutant neurons exhibit shorter and less complex dendrite arbors compared to neurons from wild-type mice (Rosso et al., 2005). These results demonstrate that Dvl1 is required for normal dendritic development in hippocampal neuron (Rosso et al., 2005). Further analyses revealed that Wnt7b/Dvl signaling regulates dendritic development through a non-canonical pathway, since activation of GSK-3β or inhibition of β-catenin is not involved. In contrast, Wnt7b and Dvl modulate dendrite development through changes in the activity of Rho GTPases and JNK. Wnt7b and Dvl activate endogenous Rac and its downstream effector JNK, this effect is abolished by Sfrp1. Moreover, inhibition of JNK or expression of a Rac dominant negative mutant in neurons blocks the Dvl function in dendritic development. These finding suggested that Dvl functions as a molecular link between Wnt factors and the cytoskeletal modulators Rho GTPases to control dendritic development (Rosso et al., 2005). In addition, a recent study shows that the non-canonical Wnt signaling is necessary for normal morphological maturation of olfactory bulb (OB) interneurons (Pino et al., 2011). Interestingly, traditionally non-canonical Wnt ligands Wnt5a and Wnt7b, but not canonical Wnts, are expressed by OB interneurons themselves (Shimogori et al., 2004). Moreover, evidence from the Wnt5a knockout mouse indicates that normal morphological development of OB interneurons is disrupted in the absence of endogenous Wnt5a, while cell number and OB architecture remain intact (Pino et al., 2011). These data are in accordance with those in hippocampal neurons where Sfrp1 blocks neurite outgrowth and non-canonical Wnt ligand Wnt7b increases dendrite complexity (Rosso et al., 2005; Endo et al., 2008).

The role of Wnt ligands on neuronal maturation have also been described in another model, such as *C. elegans* in which five Wnts were identified (Park and Shen, 2012). A recent study in *C. elegans* shows that dendrite outgrowth can be regulated by distinct processes which are independent of axon formation (Kirszenblat et al., 2011). This work suggests that the Wnt ligand (LIN-44), and its Fz receptor (LIN-17), regulate dendrite development of the oxygen sensory neurons (PQR) (Kirszenblat et al., 2011). In lin-44 and lin-17 mutants, neurons show a delayed growth. Additional experiment revealed that LIN-44 functions as an attractive cue to define the outgrowth of the dendrite (Kirszenblat et al., 2011). LIN-44 acts at very early stages of PQR development by regulating proper formation of the growth cone and its extension.

Taken together, these evidences reveal the important role for Wnt signaling pathways in regulating dendrite development and complexity and how Wnt expression and secretion may be influenced by extracellular cues to modulate neuronal morphogenesis.

### Wnt SIGNALING AT CENTRAL SYNAPSES

Wnt ligands have been linked to the assembly of structural components in presynaptic compartments. In the cerebellum, Wnt7a is expressed in granular cells at the same time as the mossy fiber axon, which is the presynaptic contact (Hall et al., 2000). Several changes remodel the connectivity between both areas to increase the contact surface. Wnt7a induces axonal spreading and incremental growth of cone size and branching, leading to the accumulation of synaptic proteins (Hall et al., 2000; Budnik and Salinas, 2011). Wnt7a probably contributes to the formation of active zones because it increases the clustering of synapsin I, a protein located in the presynaptic membrane involved in synapse formation and function (Hall et al., 2000). This effect has been blocked by the Wnt scavenger Sfrp and a mutant mice deficient in Wnt7a shows a delayed synaptic maturation (Hall et al., 2000). Then in the cerebellum, Wnt7a can act as a retrograde signal from granular cells to induce presynaptic differentiation in mossy fiber, working as a synaptogenic factor (Hall et al., 2000; Ahmad-Annuar et al., 2006). Like Wnt7a, Wnt7b, and Wnt3a increase the number of presynaptic puncta suggesting a role for these ligands in presynaptic assembly (Ahmad-Annuar et al., 2006; Cerpa et al., 2008; Davis et al., 2008). Wnt7a also increases the clustering of presynaptic proteins such as synaptophysin, synaptotagmin, and synaptic vesicle protein 2 (SV2), but does not affect postsynaptic clustering of proteins like postsynaptic density protein-95 (PSD-95; Cerpa et al., 2008). Despite Wnt7a clustering induction correlates with β-catenin stabilization, this does not involve Wnt gene target expression – an effect that is also mimicked by Wnt3a. Unexpectedly, GSK-3β is also not required for presynaptic clustering induced by Wnt7a, suggesting that an upstream mechanism is involved (Cerpa et al., 2008). It has been suggested that Wnt7a requires Dvl1 to mediate the normal recycling rate of synaptic vesicles, and the deficiency of both proteins (double null mutant) significantly reduces miniature exitatory postsynaptic current (mEPSCs) frequency, an indication of a defect in neurotransmitter release (Ahmad-Annuar et al., 2006). Additionally, the use of FM dyes has shown that Wnt7a stimulates recycling and accelerates exocytosis of synaptic vesicles (Ahmad-Annuar et al., 2006; Cerpa et al., 2008). Moreover, Wnt7a increases the frequency of mEPSCs, suggesting that Wnt7a increases the dynamic of neurotransmitter release (Ahmad-Annuar et al., 2006; Cerpa et al., 2008). Furthermore, Wnt7a/Dvl1 double mutant mice exhibit reduced mEPSC frequency at the mossy fiber-granule cell synapses, revealing a defect in neurotransmitter release as a consequence of this mutation (Ahmad-Annuar et al., 2006). Electrophysiological recordings on hippocampal rat slices also show that, in the CA3–CA1 synapse Wnt7a, but not Wnt5a, increases the amplitude of field excitatory postsynaptic potentials (fEPSP) and decreases the rate of paired pulse facilitation (PPF; Cerpa et al., 2008), a protocol used to distinguish the involvement of the presynaptic from the postsynaptic terminal. In addition, a similar modulation has been shown with nanomolar concentrations of Wnt3a, which modulates the recycling and exocytosis of synaptic vesicles in hippocampal synapses, increasing the frequency of mEPSC through a mechanism that involves Ca^2+^ entrance from extracellular media (Cerpa et al., 2008; Avila et al., 2010). Most of the ligands that are able to modulate presynaptic differentiation have shown to activate the Wnt/β-catenin signaling pathway.

Wnt7a has been also involved in trafficking of receptors, increasing the number and size of co-clusters of presynaptic α_7_-nicotinic acetylcholine receptors (α_7_-nAChR) and APC in hippocampal neurons, as well as in the modulation of the α_7_-nAChR trafficking to the nerve terminal (Farias et al., 2007), indicating that Wnt pathway components are actively involved in the functional availability of receptors in the synaptic terminal.

**FIGURE 3:**
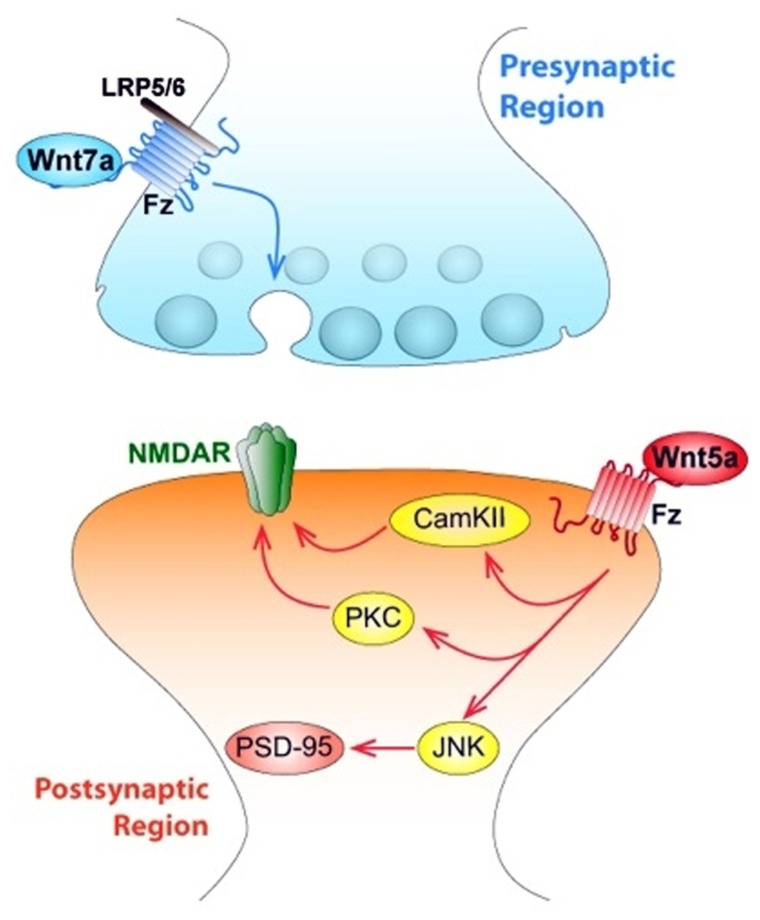
**Wnts modulate both pre- and postsynaptic regions of central synapses affecting synaptic wiring and plasticity.** The figure indicates a central synapse of the mammalian nervous system. At the *presynaptic region*, canonical Wnt7a ligand activates Fz receptor associated to LRP5/6, and triggers the activation of the synaptic vesicle cycle, which determines the release of the neurotransmitter glutamate. At *the postsynaptic level*, Wnt5a ligand activates both the Wnt/PCP (JNK) and the Wnt/Ca signaling pathways, through activation of JNK, Wnt5a regulates the clustering of PSD-95. Wnt5a also activates both non-canonical pathways that allow an increase in the traffic of NMDA receptors.

Wnt signaling also plays relevant roles in the postsynaptic structure. Wnt5a induced a transient formation of dendritic protrusions, which results in a net increase of mature dendrite spines. Video microscopy revealed that Wnt5a induced *de novo* formation of dendrite spines and also increased the size of the preexistent ones (Varela-Nallar et al., 2010). Interestingly, treatment with the soluble CRD region of Fz2, which act as a Wnt scavenger, decreases spine density in cultured neurons, supporting the idea that Wnt ligands participate in dendrite spine morphogenesis (Varela-Nallar et al., 2010). Wnt5a also induces an increase of calcium in synaptic puncta of neurons, suggesting the activation of the Wnt/Ca^2+^ signaling pathway in cultured hippocampal neurons through a mechanism that involves fast phosphorylation of CamKII induced by Wnt5a (Varela-Nallar et al., 2010), as we demonstrated previously (Farias et al., 2009). Wnt7a is also able to increase the density and maturity of dendritic spines through a CamKII-dependent mechanism (Ciani et al., 2011). Wnt7a rapidly activates CamKII in spines and inhibition of this kinase abolishes the effects of Wnt7a on spine growth and excitatory synaptic strength. These findings implicate the Wnt/Ca^2+^ signaling cascade in synaptic effects of Wnt ligands (**Figure 3**).

Interestingly, Dvl expressed only in postsynaptic spines and not in innervating presynaptic axons is enough to induce spine growth, suggesting that it is the activation of postsynaptic Wnt signaling which induces spine maturation (Ciani et al., 2011). Moreover, Dvl promotes the assembly of pre- and postsynaptic structures at pre-existing spines because this does not change the number of spines (Ciani et al., 2011). This evidence supports the idea that an extracellular signal such as Wnt7a can generate a divergent intracellular product, using a common molecule such as Dvl to support processes like synaptic differentiation (Gao and Chen, 2010), and a new role for Wnt7a inducing the formation and function of excitatory synapses through CamKII (**Figure 3**).

Wnt5a modulates postsynaptic assembly increasing the clustering of the PSD-95 via Wnt/JNK signaling pathway (Farias et al., 2009), inducing a fast increase in the number of PSD-95 clusters without affecting total levels of PSD-95 protein or presynaptic protein clustering in hippocampal cultured neurons (Farias et al., 2009; **Figure 3**). PSD-95 is a scaffold protein of the postsynaptic density, which is a multiprotein complex that interacts with key molecules involved in the regulation of glutamate receptor targeting and trafficking and regulatory proteins relevant for neurotransmission (Han and Kim, 2008). When hippocampal neurons were incubated with the formylated hexapeptide Foxy-5, derived from the sequence of Wnt5a and mimics the full Wnt5a action, there was an increase in PSD-95 since 1 h, but after 24 h an increase in the SV2 clustering was also observed. In consequence, there was an increase in the total number of synaptic contacts (Varela-Nallar et al., 2012).

At the neuromuscular junction of vertebrate skeletal muscles, Wnt3 was also able to induce recruitment of AChRs (Henriquez et al., 2008). This effect requires Dvl1 and agrin, a protoglycan released by motoneurons, but does not involve the Wnt/β-catenin pathway. Instead, aggregation is induced through activation of Rac1 (Henriquez et al., 2008). However, Wnt3a inhibits agrin-induced AChR clusters through the activation of the Wnt/β-catenin pathway, suggesting that Wnt signaling dynamically regulates the interaction between postsynaptic components during the establishment of neuromuscular junctions (Wang et al., 2008).

Different Wnts have shown modulatory effects on glutamatergic neurotransmission. Wnt3a modulates the recycling of synaptic vesicles in hippocampal synapses (Cerpa et al., 2008; Varela-Nallar et al., 2009) and is able to induce an increase in the frequency of mEPSCs (Avila et al., 2010). In hippocampal slices, blockade of Wnt signaling impairs long-term potentiation (LTP), whereas activation of Wnt signaling facilitates LTP (Chen et al., 2006). In the case of Wnt5a, acute application of this ligand in hippocampal slices increases the amplitude of fEPSP and upregulates synaptic *N*-methyl-D-aspartate (NMDA) receptor currents facilitating induction of LTP (Cerpa et al., 2010, 2011; Varela-Nallar et al., 2010). Interestingly, Wnt5a produced a two-step increase in the amplitude of NMDA receptor responses, not α-amino-3-hydroxy-5-methyl-4-isoxazolepropionic acid (AMPA) receptors (Cerpa et al., 2011). There is a fast PKC-dependent potentiation and a slower JNK-dependent potentiation that does not require previous activation of PKC (Cerpa et al., 2011).

Wnt5a also regulates postsynaptically the hippocampal inhibitory synapses (Cuitino et al., 2010). Wnt5a induces surface expression and maintenance of GABA_A_ receptor in the membrane of hippocampal neurons, increases the amplitude of gamma-aminobutyric acid (GABA)-currents due to postsynaptic mechanisms, and induces the recycling of functional GABA_A_ receptors through activation of CamKII (Cuitino et al., 2010). Therefore Wnt5a is able to modulate both, excitatory and inhibitory synapses which must be relevant for neurotransmission.

## ROLE OF Wnt SIGNALING IN ALZHEIMER’S DISEASE

Major neurological diseases are all progressive disorders with common symptoms: a range of neuropsychiatric features, massive neuronal degeneration, and neither preventive nor effective long-term treatment strategies available. However, all of them develop in particular brain regions generating a specific phenotype according to the circuit that is being affected. Here, we review recent studies related to the progression of AD in which the Wnt signaling pathway and its components might be relevant. AD is a neurodegenerative disorder characterized by progressive deterioration of cognitive functions, caused by synaptic dysfunction and damage of specific brain regions (Mattson, 2004; Toledo et al., 2008). Distinctive features of AD brains are the presence of senile plaques, composed by extracellular deposits of amyloid-β (Aβ) peptides and neurofibrillary tangles (NFTs), composed by intracellular aggregates of hyper-phosphorylated tau protein (Mayeux and Stern, 2012). Oligomeric forms of Aβ_1-42_ are the physiologically relevant neurotoxic Aβ species and Aβ oligomers isolated from AD brains can damage the memory and alter hippocampal synaptic plasticity in healthy rats, inhibiting LTP, increasing long-term depression and reducing spine density in the hippocampus *in vivo* (Shankar et al., 2007). The presence of Aβ oligomers reduces the fEPSP and EPSCs, but not the PPF in the CA3–CA1 synapse of rat hippocampal slices (Cerpa et al., 2010; Inestrosa et al., 2012). Altogether these evidences suggest that AD cognitive decline might be due to a direct effect of Aβ oligomers on synaptic transmission (Palop and Mucke, 2010).

During more than a decade, a strong relationship between an impaired Wnt signaling pathway activity and neuronal damage in AD has been raised (De Ferrari and Inestrosa, 2000; Inestrosa et al., 2000; Garrido et al., 2002; De Ferrari et al., 2003; Inestrosa and Arenas, 2010). Different studies have shown that Wnt signaling components are altered in AD (Zhang et al., 1998; Inestrosa et al., 2002; Caricasole et al., 2004; Ghanevati and Miller, 2005; De Ferrari et al., 2007; Magdesian et al., 2008). Among the Wnt components that are affected in AD, it was shown that β-catenin levels are reduced in AD patients carrying presenilin-1-inherited mutations (Zhang et al., 1998), while the secreted Wnt antagonist Dkk1 is elevated in postmortem AD brains and brains from transgenic mouse models for AD (Caricasole et al., 2004; Rosi et al., 2010). A variant of the LRP6 has been associated with late-onset AD, which confers low levels of Wnt signaling (De Ferrari et al., 2007). Epidemiological data show an increased risk for AD in populations where the allele 4 of apo-lipoprotein E (apoE4) is present. Interestingly apoE4 causes inhibition of the canonical Wnt signaling in PC12 cells upon stimulation with Wnt7a as determined by luciferase activities and nuclear β-catenin levels (Caruso et al., 2006). A direct Aβ binding to the extracellular CRD-Fz5 receptor at or in close proximity to the Wnt-binding site inhibits the canonical Wnt signaling pathway (Magdesian et al., 2008), linking directly Aβ to Wnt impairment. Moreover, the exposure of cultured rat hippocampal neurons to Aβ results in inhibition of canonical Wnt signaling as determined by destabilization of endogenous levels of β-catenin, increase in GSK-3β activity, and a decrease in the expression of some Wnt target genes (Alvarez et al., 2004). Chronic overexpression of Dkk-1 causes age-related tau phosphorylation and cognitive deficits (Killick et al., 2012). During Aβ exposure there is induction of Dkk-1 expression that depends on p53 (Killick et al., 2012). This generated Dkk-1 can bind LRP5/6 to inhibit their interaction to Wnts. Thus Wnt cannot inhibit GSK-3β facilitating tau hyperphosphorylation and NFT formations, leading to neurotoxicity and apoptosis caused by Aβ peptides (Caricasole et al., 2004). The use of an antibody antiDkk-1 also blocks the synaptic loss induced by Aβ (Purro et al., 2012). In the same report, Purro et al. (2012) documented that Dkk-1 can reversibly reduce the amount of synaptic proteins and the number of active presynaptic sites, by inducing synaptic disassembly at pre- and postsynaptic sites and not by degrading proteins.

Recently, new evidence supports a link between Aβ toxicity and the Wnt pathway. Aβ substantially increases the intracellular protein clusterin, which induces Dkk-1 expression (Killick et al., 2012). The C-terminal of the Dkk-1 protein, which antagonizes canonical Wnt pathway binding LRP5/6, later activates gene transcription involved in AD-like pathology (Killick et al., 2012). Because Dkk-1 blocks Wnt/β-catenin, Dkk-1 activates the Wnt/JNK pathway, as has been shown from the increase in c-Jun activity (Killick et al., 2012). Thus, the transcriptional Aβ effects occur because Dkk-1 activates Wnt/JNK pathway. Clusterin has been recently identified as a susceptibility factor in late-onset AD (Harold et al., 2009; Lambert et al., 2009) and several genes from Wnt/JNK have been found in the AD human brain (Killick et al., 2012).

Synaptic failure is an early event in AD, and soluble Aβ oligomers are proposed to be responsible for the synaptic pathology that occurs before the plaque deposition and neuronal death (Serrano-Pozo et al., 2011). Electrophysiological analysis of Schaffer collaterals-CA1 glutamatergic transmission in hippocampal slices demonstrated that Wnt5a prevents the decrease in the amplitude of fEPSP and EPSCs induced by Aβ oligomers, indicating that Wnt5a prevents the synaptic damage triggered by Aβ (Cerpa et al., 2010). Moreover, Wnt5a prevents the decreases in PSD-95 and synaptic loss in cultured hippocampal neurons (Farias et al., 2009) (Cerpa et al., 2010), supporting that Wnt5a improves synaptic function in the presence of Aβ.

**FIGURE 4:**
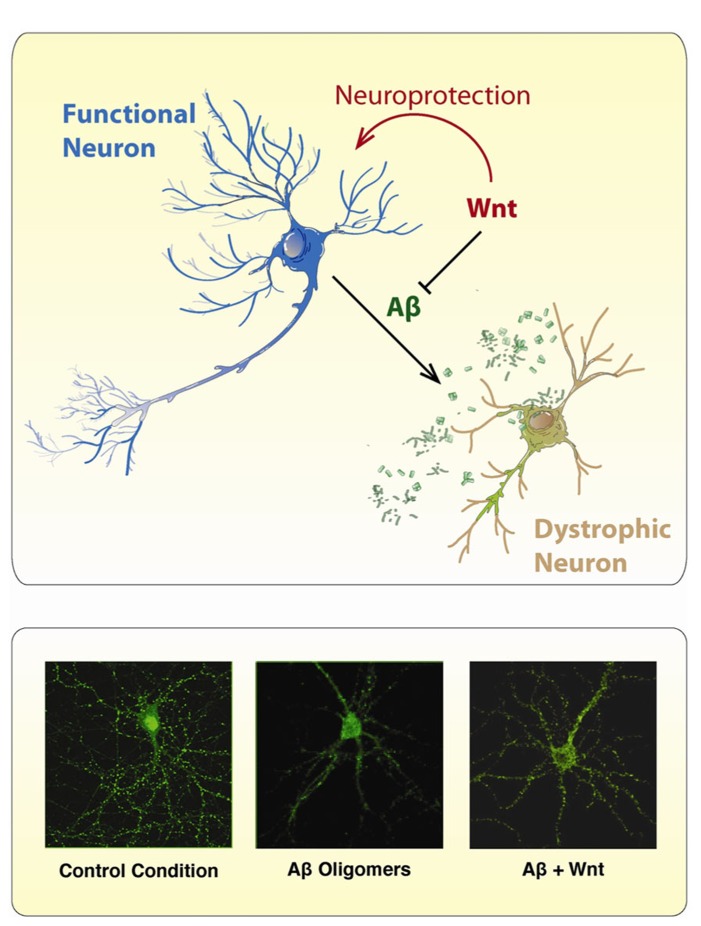
**Wnt ligands protect neurons from the toxic effects of Aβ Oligomers.** The scheme illustrates the neurotoxic effect of Aβ oligomers on neurons which became dystrophic showing neurite fragmentation and eventual cell death. However, in the presence of a canonical Wnt ligand, neurons became neuroprotected and no neuropathological changes are evident. The immunofluorescence photographs presented below, were taken against the *GFP* expressing protein present in hippocampal neurons incubated under different conditions: (1) control conditions, (2) Aβ oligomers, and (3) Aβ oligomers plus Wnt7a. In the presence of Aβ oligomers, treated neurons showed typical neurodegenerative alterations including loss of somatodendritic arborization and neurite fragmentation. After co-incubation with Wnt7a, the neurotoxic effect of Aβ oligomers is not longer present, and hippocampal neurons look pretty normal.

Several studies have shown neuroprotective properties of the Wnt signaling activation against the toxicity of Aβ peptide (**Figure 4**). The protective effect of Wnt3a against the toxicity of Aβ oligomers was shown to be mediated by the Wnt Fz1 receptor, since this effect is modulated by the expression levels of Fz1 in both, PC12 cells and hippocampal neurons (Chacon et al., 2008). Overexpression of Fz1 significantly increased cell survival induced by Wnt3a and diminished caspase-3 activation, while knocking-down the expression of the receptor by antisense oligonucleotides decreased the stabilization of β-catenin induced by Wnt3a and decreases the neuroprotective effect elicited by this Wnt ligand (Chacon et al., 2008). These studies support the evidence that alterations in Wnt/β-catenin are involved in AD.

One of the hallmarks of AD brains is the abnormal phosphorylation of the tau protein which accumulates as intraneuronal NFT (Serrano-Pozo et al., 2011). Cultured neurons exposed to Aβ show an increase in GSK-3β activity (Takashima et al., 1993; Alvarez et al., 2004; Inestrosa et al., 2005). Importantly, active GSK-3β has been found in brains staged for AD neurofibrillary changes with a concomitant decreases in β-catenin levels and an increase in tau hyperphosphorylation (Pei et al., 1999). Also, neurodegeneration and spatial learning deficits have been observed in GSK-3β conditional transgenic mice (Lucas et al., 2001; Hernandez et al., 2002). Moreover, over-expression of GSK-3β in mice prevents the induction of LTP and reduces spatial learning (Hernandez et al., 2002; Hooper et al., 2007), linking the characteristic memory failure in AD to the increase in GSK-3β. In cultured neurons the toxicity mediated by Aβ depends on increased GSK-3 activity, and reverses when GSK-3β expression or activity is blocked (Busciglio et al., 1995; Alvarez et al., 1999). We previously demonstrated that activation of Wnt signaling inhibits GSK-3β and leads to neuroprotection in both hippocampal cultured neurons and *in vivo* transgenic model of AD (Alvarez et al., 2004; Quintanilla et al., 2005; Toledo and Inestrosa, 2010). Moreover, Li et al. (2007) found that tau phosphorylation, which inhibits competitive phosphorylation of β-catenin by GSK-3β, protects neurons from apoptosis. This results support a role of β-catenin as a survival element in AD. Finally, the activation of several signaling pathways that crosstalk with the Wnt pathway, including the nicotinic and muscarinic ACh receptors, peroxisome proliferator-activated receptor (PPAR) α and γ, anti-oxidants, and anti-inflammatory pathways, all support the neuroprotective potential of the Wnt cascades in AD (Inestrosa and Toledo, 2008; Inestrosa and Arenas, 2010; Inestrosa et al., 2012).

## CONCLUSION

This review focused on the role of Wnt proteins on neuronal development and their participation on the synaptic function. Wnt signaling regulates neuronal maturation by stimulation of dendrite formation and complexity. Particularly, it has been reported that neurons exposed to Wnt elicit longer dendrites and more complex dendritic branches. In addition, several works have demonstrated that the non-canonical Wnt pathways would modulate dendrite formation. Thus, Wnt effectors as JNK and CaMKII may control dendrite architecture and neuronal maturation (Threadgill et al., 1997; Rosso et al., 2005; Wayman et al., 2006). Importantly, these effectors have been implicated in cytoskeletal remodeling by controlling MAPs phosphorylation and microtubule dynamics and, they also bind and modulate actin filopodia extension (Shen et al., 1998; Chang et al., 2003; Fink et al., 2003; Bjorkblom et al., 2005). Changes in the organization and stability of cytoskeletal components by Wnt pathways likely affect dendritic growth and dynamics. Furthermore, neuronal activity plays a central role in dendrites formation and maintenance. Several studies have shown that the stimulation of neuronal activity leads to increase the expression and/or secretion of Wnt proteins and also can modify the MAPs activity and microtubule stability (Vaillant et al., 2002; Yu and Malenka, 2003; Wayman et al., 2006). Dendrites morphologies influence on synaptic function and neuronal circuits formation. Thus, the timing of synapse formation coincides with the period of dendritic growth and branching. A great body of evidences extensively suggests that Wnt signaling modulate synaptic function and plasticity. Thus, the pre- and postsynaptic terminal assembly is modulated by Wnt signaling to maintain the central connectivity (Hall et al., 2000; Packard et al., 2002; Ahmad-Annuar et al., 2006; Cerpa et al., 2008; Henriquez et al., 2008; Farias et al., 2009; Cuitino et al., 2010). Taken together, these compiled findings provide important insights about the involvement of Wnt signaling pathways on the formation and functioning of neuronal circuits.

## Conflict of Interest Statement

The authors declare that the research was conducted in the absence of any commercial or financial relationships that could be construed as a potential conflict of interest.
